# Factors influencing UK arthroplasty surgeons' decision‐making between total and medial unicompartmental knee surgery: A vignette‐based behavioural experiment

**DOI:** 10.1002/jeo2.70178

**Published:** 2025-02-28

**Authors:** Martine Nurek, Omar Musbahi, Martinique Vella Baldacchino, Robert Hamm, Caroline B. Hing, Justin Cobb, Olga Kostopoulou, UNITES Consortium, Andrew Ng, Muhammad Adeel Akhtar, Adrian J. Cassar‐Gheiti, Alan Norrish, Alasdair J. A. Santini, Amit R. Patel, Benedict Lankester, Ben Waterson, Chloe E. H. Scott, David Houlihan‐Burne, Reza Mansouri, Manish Kiran, Ghias Bhattee, Jonathan Quayle, Jonathan Phillips, James R. A. Smith, Keshav Mathur, Moez Zeiton, Nathanael Ahearn, Nigel Rossiter, Nick Ohly, Nick London, Pregash Ellapparadja, Rahul S. Kotwal, Rahul Bhattacharyya, Reshid Berber, Robert Walker, Ravikumar Pydisetty, Sean O'leary, Simon B. Barton, Simon P. White, Stefan Bajada, Sujit Agarwal, William F. M. Jackson, Yuvraj Agrawal, Zameer Shah, Andrew Toms, Amit Bishnoi

**Affiliations:** ^1^ Department of Surgery and Cancer Imperial College London London UK; ^2^ MSk Lab, Sir Michael Uren Hub Imperial College London London UK; ^3^ University of Oklahoma Oklahoma USA; ^4^ St George's Hospital NHS Foundation Trust London UK; ^5^ Pinderfields General Hospital Wakefield UK; ^6^ NHS Fife Kirkcaldy UK; ^7^ National Orthopaedic Hospital Cappagh Dublin Ireland; ^8^ University of Nottingham Nottingham UK; ^9^ Liverpool University Hospitals Liverpool UK; ^10^ Royal Stoke University Hospital Stoke‐on‐Trent UK; ^11^ Somerset Foundation Trust Somerset UK; ^12^ Royal Devon and Exeter Hospital Exeter UK; ^13^ Royal Infirmary of Edinburgh Edinburgh UK; ^14^ Fortius Clinic London UK; ^15^ Frimley Health NHS Frimley UK; ^16^ Dartford and Gravesham NHS Trust Dartford UK; ^17^ London North West University Healthcare NHS Trust London UK; ^18^ Salisbury NHS Trust Salisbury UK; ^19^ Princess Elizabeth Orthopaedic Centre Exeter UK; ^20^ University Hospitals Bristol and Weston NHS Trust Bristol UK; ^21^ Worcestershire NHS Trust Worcester UK; ^22^ Royal Bolton Hospital Farnworth UK; ^23^ South Devon and Torbay NHS Trust Torquay UK; ^24^ Basingstoke NHS Trust Basingstoke UK; ^25^ NHS Golden Jubilee Clydebank UK; ^26^ Yorkshire Knee Clinic Leeds UK; ^27^ Tameside and Glossop Integrated Care NHS Foundation Trust Ashton‐under‐Lyne UK; ^28^ Cwm Taf Morgannwg University Health Board Abercynon UK; ^29^ NHS Lanarkshire University Hospital's Wishaw UK; ^30^ Nottingham University Hospitals Nottingham UK; ^31^ Royal Cornwall Hospitals NHS Trust Truro UK; ^32^ Whiston Hospital Rainhill UK; ^33^ Royal Berkshire NHS Foundation Trust Reading UK; ^34^ Wrightington Orthopaedic Hospital Lancashire UK; ^35^ University Hospital of Wales Cardiff UK; ^36^ Glangwili Hospital Carmarthen UK; ^37^ Midland Metropolitan University Hospital Smethwick UK; ^38^ Nuffield Orthopaedic Centre Oxford UK; ^39^ The Royal Orthopaedic Hospital NHS Trust Birmingham UK; ^40^ Guy's & St. Thomas' Hospitals London UK; ^41^ University Hospitals of Leicester NHS Trust Leicester UK

**Keywords:** decision‐making, knee arthroplasty, osteoarthritis, total knee arthroplasty, unicompartmental

## Abstract

**Purpose:**

Surgical options for end‐stage knee osteoarthritis (OA) include total and medial unicompartmental knee replacement (TKR and UKR). Deciding which surgery to perform is complex and ill‐defined, yet it has important implications for patients and the health service. The study aimed to identify clinical and surgeon factors predicting surgeons' preferences.

**Methods:**

Based on a preliminary survey of 162 UK surgeons, we identified clinical features frequently considered when deciding between TKR and UKR. By systematically varying patient age, obesity, site of pain, anaesthetic risk and anterior cruciate ligament (ACL) integrity, we constructed 32 clinical vignettes. We used these in a new survey, where surgeons indicated which surgery they would recommend on an 11‐point rating scale with end points anchored at ‘definitely TKR’ and ‘definitely medial UKR’. Data were analysed with mixed‐effects linear regressions.

**Results:**

Eighty‐three UK arthroplasty surgeons completed the vignettes. Preference for UKR over TKR was significantly lower for patients over 50 years (*b* = −0.57 [−0.82 to −0.33], *p* < 0.001) with abnormal ACL (*b* = −1.93 [−2.17 to −1.68], *p* < 0.001) and severe systemic disease (*b* = −0.46 [−0.70 to −0.21], *p* < 0.001). Obesity was a weak and unreliable predictor, and we did not detect any influence of site of pain. The surgeons' habitual practice (proportion of UKRs over all knee replacements performed in a typical year) was the second strongest predictor after ACL (*b* = 1.26 [0.54–1.99], *p* = 0.001).

**Conclusions:**

ACL integrity was the most important determinant of surgeons' preferences between TKR and UKR. Their habitual practice was also a strong predictor, outweighing most clinical factors in the vignettes.

**Level of Evidence:**

Level II, prospective cohort study.

**Abbreviations**
ACL: anterior cruciate ligament
AOT: Actively Open‐minded Thinking
ASA: American Society of Anaesthesiologists
BASK: British Association for Surgery of the Knee
BMI: body mass index
NICE: National Institute for Health and Care Excellence
NIHR: National Institute for Health and Care Research
OA: osteoarthritis
TKR: total knee replacement
UKR: unicompartmental knee replacement

## BACKGROUND

Knee osteoarthritis (OA) is the most common form of arthritis, affecting an estimated 16% of the global population [10]. The lifetime risk of symptomatic knee OA has been estimated to be between 14% and 45% [28, 32]. Knee OA is also a leading source of chronic pain and disability in developed nations, ranked as the 11th largest contributor to global disability according to the Global Burden of Disease Study [9, 45]. The high prevalence and incidence of knee OA make it a major public health concern with the Framingham study finding an increase in the prevalence of symptomatic knee OA of 4.1% in women and 6% in men [35].

If non‐surgical treatments such as medication, physical therapy and weight loss are not effective, knee replacement surgery may be considered. The UK Clinical Research Practice Datalink database estimates a lifetime risk of knee replacement surgery of 8.1%–10.8% in the UK population over 50 [11]. Currently, over 100,000 knee replacement procedures are performed in the United Kingdom in each calendar year and, with the projected annual incidence of knee OA increasing from 43 to 133 per 100,000 people, joint replacement surgery is expected to increase [31, 34, 41]. Joint replacement surgery options include total knee replacement (TKR) and unicompartmental knee replacement (UKR). There is good evidence that UKR offers better function, quicker recovery, and fewer complications [42, 46, 48], as well as significant cost savings [4]; hence, the UK National Institute for Health and Care Excellence (NICE) guidelines advise that eligible patients are offered UKR when indicated [34]. According to the 20th National Joint Registry annual report, around half of knee OA patients are eligible for a UKR, nevertheless, the majority (87.7%) of all primary knee operations are TKRs and only 9.8% are medial/lateral UKRs [17, 36].

With the demand for TKR expected to grow by more than 600% by 2030, it is crucial that the correct prosthesis is performed for each patient [25]. However, the UKR versus TKR decision is complex, ill‐defined and seemingly subjective as surgeons are found to vary greatly in their treatment of patients with identical pathologies [3]. Currently, little is known about how arthroplasty surgeons decide between TKR and UKR, and it is unclear why relatively few UKRs are performed despite evidence of their better outcomes.

We therefore conducted a vignette‐based behavioural experiment to determine what information experienced UK knee surgeons consider when deciding whether to perform TKR versus UKR. Given that surgeons who perform UKR more frequently (40%–60% of their caseload) have better outcomes than those who do so less frequently (less than 5% of their caseload) [19], we also explored the influence of surgeon factors on decision‐making: years of experience, habitual practice, proportion of work conducted in independent practice, and thinking style. Thinking style was measured using the 11‐item ‘Actively Open‐minded Thinking’ (AOT) scale [2, 16]. AOT measures people's tendency to question their own thinking and beliefs by, for example, seeking disconfirming information, adopting various perspectives, and considering alternatives. The AOT scale has been used before in vignette‐based behavioural experiments on the diagnostic reasoning of UK General Practitioners. GPs higher in AOT indicated lower certainty about an initial diagnostic hypothesis that was based on limited information and engaged in more extensive search for information before reaching a final conclusion [23].

## METHODS

### Survey for vignette design

In March–April 2021, the British Association for Surgery of the Knee (BASK) sent an invitation email to its members (UK knee surgeons) containing a link to an anonymous online survey hosted by Qualtrics (Qualtrics XM). Participants read information about the survey and provided informed consent. They were then shown a list of clinical features and were asked to select all those that they always considered when deciding between total and unicompartmental knee replacement. They were explicitly instructed not to select information that they considered only sometimes. They were also asked to name any other factors that they considered, which were not included in the list.

One hundred sixty‐two surgeons completed the survey: 98% consultants, 69% fellowship‐trained in UKR, a median of 26% of work conducted in private practice (mean 31%, standard deviation [SD] 28%, range 0%–100%), a median of 72 TKRs (mean 69, SD 29, range 8–100) and 23 UKRs (mean 26, SD 22, range 0–100) performed in the last year.

Table 1 shows the features that were presented to surgeons and the frequency with which each feature was selected. Table 2 shows the most common features that surgeons generated and the frequency with which each was generated.

**Table 1:** Clinical features presented to 162 surgeons in the preliminary survey with selection rate (% and frequencies), and our final decision to include or exclude from vignette design and to keep constant or manipulate.

Item	%	Count	Vignette decision
**Items relating to patient history:**			
Inflammatory disease	83%	135/162	Include, keep constant (no history of rheumatoid inflammatory disease)
Patient preferences regarding total vs. unicompartmental knee replacement	78%	127/162	Include, keep constant (patient has no preference)
Activity demand (high or low)	40%	64/162	Include, keep constant (high activity demand)
Obesity	36%	59/162	Include, manipulate (obese vs. not)
Infection	27%	44/162	Omitted: surgery would not be offered to patients with recent infection
Anaesthetic risk (high or low)	25%	41/162	Include, manipulate (severe systemic disease vs. not)
Neuropathy	21%	34/162	Omitted as infrequently selected
Vascular disease	14%	23/162	Omitted as infrequently selected
COVID‐19 risk level (high or low)	7%	11/162	Omitted as infrequently selected
**Items relating to examination:**			
Inflammatory status (rheumatoid vs. osteoarthritis)	89%	144/162	Include, keep constant (no history of rheumatoid inflammatory disease)
Medial/lateral collateral ligaments	87%	141/162	Include, keep constant (no collateral injury)
Anterior/posterior cruciate ligaments	86%	139/162	Include, manipulate ACL (normal vs. abnormal), keep PCL constant (PCL intact)
Site of wear—if selected, surgeons were asked to specify the relevant site/s, by ticking all that apply:	79%	128/162	
Medial tibial plateau	65%	106/162	Included as part of x‐ray
Lateral femoral condyle	64%	104/162	Include, keep constant (mild cartilage fibrillation: lateral femoral condyle)
Medial femoral condyle	63%	102/162	Included as part of x‐ray
Lateral tibial plateau	63%	102/162	Include, keep constant (no loss of joint space on lateral side)
Lateral patellar facet	59%	96/162	Include, keep constant (no further information could be obtained from skyline view)
Lateral trochlear groove	54%	88/162	Include, keep constant (no further information could be obtained from skyline view)
Medial patellar facet	33%	53/162	Include, keep constant (mild cartilage fibrillation: medial patellar facet)
Medial trochlear groove	32%	51/162	Include, keep constant (no further information could be obtained from skyline view)
**Items relating to X‐ray:**			
Loss of space on the lateral side in AP or Rosenberg	84%	136/162	Include, keep constant (there is no loss of space on the lateral side on AP or Rosenberg)
Location of wear (patellofemoral joint vs. medial/lateral)	81%	131/162	Include, keep constant (no loss of space on lateral side; no further information could be obtained from skyline views)
Extent of wear patch on AP and Rosenberg (bone‐on‐bone vs. not)	65%	105/162	Include, keep constant (extent of wear patch on AP and Rosenberg: Ahlback Grades III and IV medial tibiofemoral joint)
Anterior tibial subluxation on the lateral view	56%	90/162	Include, manipulate (ACL normal vs. abnormal)
PFJ arthrosis on medial side only	38%	61/162	Omitted as infrequently selected
Opening up of the medial side of fluoroscope available for all	17%	27/162	Omitted as infrequently selected

*Note*: Blue shading indicates that the item was included in the vignettes and held constant; yellow shading indicates that the item was included in the vignettes and varied; no shading indicates that the item was excluded from the vignettes.

Abbreviations: AP, anterior‐posterior; PCL, posterior cruciate ligament.

**Table 2:** Clinical features generated by surgeons in the preliminary survey.

Item	%	Count	Vignette decision
Site of pain, as perceived by patient	30%	21/69	Include, manipulate (generalised vs. medial)
Range of motion	17%	12/69	Include, keep constant (normal—no fixed flexion deformity)
Patient age	16%	11/69	Include, manipulate (<50 vs. 50+)
MRI scan	14%	10/69	Include, keep findings constant

*Note*: Blue shading indicates that item was included in the vignettes and held constant; yellow shading indicates that item was included in the vignettes and varied.

Abbreviation: MRI, magnetic resonance imaging.

These responses were used to design the vignettes for the main study. Specifically, features that were selected infrequently by surgeons were omitted from the vignettes, while commonly selected features were either kept constant or were varied in the vignettes. We chose to vary relatively controversial features while keeping more accepted features constant; this was based on evidential review and team discussions [3, 5, 21, 39]. Specifically:

- *Obesity* was varied (present vs. absent) as a recent systematic review has shown that while BMI is not a contra‐indication for UKR, obese patients may have a higher risk of aseptic failure of UKR [6]. The influence of obesity on surgeons' UKR/TKR decision‐making was thus deemed worthy of further investigation.
- *Anaesthetic risk* was varied as it remains controversial in deciding whether to perform a UKR or TKR [6, 26].
- *ACL integrity* was varied as the literature has shown controversy in utilising UKR in the presence of a ruptured ACL [3, 5, 47].
- *Site of pain* and *patient age* were surgeon‐generated features and were varied because previous studies on a smaller number of surgeons have shown that surgeons do not concur regarding patients with the same pathology or age [3, 4, 21, 39].
- *Patient preference* was kept constant (no preference) as the study was intended to focus solely on surgeon preferences. We were mindful that surgeons would weigh heavily patient preferences, which could obscure more subtle effects of the clinical variables.
- *Inflammatory disease*: the vignettes described patients with no inflammatory disease since the presence of inflammatory disease would exclude patients from UKR consideration, according to the published literature [3].
- *Site of wear, activity demand, PCL integrity and collateral ligament integrity*: all were kept constant as these are accepted indications for UKR, with low activity demand patients and those with ruptured collateral ligaments more likely to require a TKR or constrained TKR implant.

### The vignettes

Thirty‐two clinical vignettes were designed depicting patients with knee damage sufficient to warrant surgery. Table 3 presents the features kept constant. Table 4 presents the features that we varied systematically across the vignettes in a full factorial design. Each of the 32 vignettes was accompanied by an anterior‐posterior (AP) and lateral knee radiograph. There were 15 radiographs in total, each of which was used approximately twice (for practical reasons). Each radiograph was consistent with the patient description in the vignette and did not contain any other decision‐relevant information over and above that contained within the vignette. To minimise response fatigue, the vignettes were divided in two sets (A and B), to be completed on different days. Half of the respondents saw set A first and the other half set B first, by random assignment. All the vignettes are presented in the Supporting Information S1: SM1.

**Table 3:** Information held constant across vignettes.

History	No history of rheumatoid inflammatory disease Activity demand is high (the patient wishes to remain active) The patient has no preference between TKR and medial UKR
Examination	Medial/lateral collateral ligaments are normal Range of motion is normal (no fixed flexion deformity)
Radiographs	Extent of wear patch on AP and Rosenberg views: Ahlback^a^ Grades III and IV medial tibiofemoral joint (equivalent to Kellgren–Lawrence Grades III and IV) No loss of space on the lateral side on AP or Rosenberg No further information determined from the skyline view
MRI	PCL intact Severe chondral damage medial femoral condyle and medial tibial plateau Mild cartilage fibrillation lateral femoral condyle and medial patellar facet

Abbreviations: AP, anterior‐posterior; PCL, posterior cruciate ligament; TKR, total knee replacement; UKR, unicompartmental knee replacement.

a Ahlbäck classification of knee osteoarthritis [1]: Grade I = joint space narrowing (<3 mm), Grade II = joint space obliteration, Grade III = minor bone attrition (0–5 mm), Grade IV = moderate bone attrition (5–10 mm), Grade V = severe bone attrition (>10 mm).

**Table 4:** Factors manipulated across vignettes.

Manipulated factors	Factor levels	Range
Age	0: < 50 years	40–80 years
	1: ≥ 50 years	
BMI	0: < 30	19–36
	1: ≥ 30	
Site of pain	0: Generalised	N/A
	1: Medial	
American Society of Anaesthesiologists Physical Classification score	0: Healthy/mild systemic disease	N/A
	1: Severe systemic disease	
Anterior cruciate ligament integrity	0: Normal	N/A
	1: Abnormal	

Abbreviation: BMI, body mass index.

### Sample size and recruitment to the vignette study

In March 2022, BASK sent another invitation email to its members containing a link to an online Expression of Interest form. The form collected the following data: year of completion of training, fellowship training in UKR, number of TKRs and UKRs performed in a typical year prior to COVID (e.g., 2019), and proportion of work conducted in independent versus public practice.

The target sample size of at least 81 arthroplasty surgeons practising in the United Kindom was selected as it afforded 80% power to detect a small‐to‐medium effect (*f* ^2^ = 0.1) [40] in a multiple linear regression measuring the effect of five predictors (clinical features) on surgeons' decision‐making (UKR vs. TKR), with *α* at 0.05 (sample size calculation conducted with G*Power 3.1).

### Procedure

After providing informed consent, surgeons were presented with task instructions. Surgeons were then presented with Table 3 and informed: ‘Please assume that this information is true for all patients. You do not need to memorise this information. It will be available for view at any time, upon request’.

**Figure 1:**
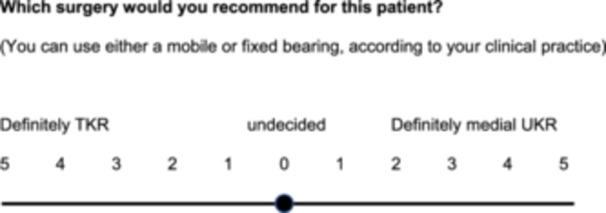
Response scale used to elicit surgical judgement per vignette. TKR, total knee replacement; UKR, unicompartmental knee replacement.

Each surgeon then had to review 16 of the 32 vignettes (set A or B), in a random order. Surgeons were asked which surgery they would recommend for the patient and responded on an 11‐point rating scale, anchored at ‘definitely TKR’ and ‘definitely medial UKR’ with ‘undecided’ in the middle (Figure 1).

Twenty‐four hours after completing the set of 16 vignettes, each surgeon was invited to review the remaining set of 16 previously unseen vignettes. They were then asked to complete the 11‐item AOT scale. For each AOT item, surgeons indicated their agreement on a 5‐point Likert scale, ranging from ‘completely disagree’ to ‘completely agree’ (see Supporting Information S1: SM2). As TKR is the more traditional option, we expected that surgeons with higher AOT scores (indicating a greater tendency to question the status quo) would be more inclined towards UKR than those lower in AOT. Data were collected between March and November 2022.

### Statistical analyses

TKR/UKR responses were measured on a scale from −5 (definitely TKR) to +5 (definitely medial UKR). Habitual practice (the self‐reported tendency to perform UKR versus TKR in clinical practice) was measured as the number of UKRs divided by the total number of knee replacements (UKR and TKR) performed in a typical year, then multiplied by 100 to form a percentage. The AOT score was calculated by reverse‐scoring 5 items and then summing responses to the 11 scale items per participant.

Using a mixed‐effects linear model, we regressed TKR/UKR responses on the five manipulated clinical features (Table 4), including a random intercept for surgeons to account for clustering. To explore the influence of surgeon variables, we then added the following predictors to the model: years of experience (number of years since training completion), fellowship training in UKR, habitual practice, proportion of work conducted in independent versus public practice, and AOT score. All surgeon variables were dichotomised (below vs. above the median) to aid comparability with the clinical features, which were binary.

To aid interpretation further, we classified responses as either ‘UKR’ (ratings to the right of the scale midpoint in Figure 1) or ‘TKR’ (ratings to the left of the scale midpoint). This dichotomised variable (UKR = 0, TKR = 1) was then regressed on the clinical features and surgeon variables, using a mixed‐effects binary logistic regression model. Ratings at the midpoint of the scale (0), showing uncertainty about or indifference between the two surgery types, were excluded from this analysis. Statistical analyses were performed using SPSS 26.0 and were confirmed using Stata/MP 17.0.

## RESULTS

### Sample demographics

Of the 578 BASK members who received the invitation email, 93 completed the first set of vignettes (16% response rate). Of these, 84 (90%) also completed the second set. The nine respondents who did not complete the second set of vignettes were excluded from the analyses, as was one respondent who was subsequently discovered to be a trainee and therefore ineligible. The final sample comprised 83 consultant arthroplasty surgeons, with a median of 12 years of experience post‐qualification (SD 8, mean 12, range 0–32).

Among the 83 respondents, 62 (75%) had fellowship training in UKR. In a typical year, the sample performed a median of 30 UKR procedures (mean 36, SD 31, range 0–160) and 80 TKR procedures (mean 88, SD 49, range 20–250); the median proportion of UKR procedures was therefore 23% (mean 29%, SD 17%, range 0%–80%). The median proportion of work conducted independently (vs. public sector) was 20% (mean 22%, SD 23%, range 0%–100%). The median AOT score was 45 (mean 45, SD 4, range 33–55) out of a possible 55.

### Surgical judgements (descriptive statistics)

The grand median for TKR/UKR responses on the 11‐point scale (−5 = definitely TKR, 0 = undecided, +5 = definitely medial UKR) was 0.5 (mean 0.8, SD 1.8, range −2.5 to 5.0), indicating a slight preference for UKR in the vignettes. When responses were categorised as either TKR (ratings to the left of the scale midpoint), UKR (ratings to the right of the scale midpoint), or undecided (ratings at the scale midpoint), the proportions were: TKR 37% (979 out of 2656), UKR 57% (1508 out of 2656), and undecided 6% (169 out of 2656). Table 5 shows the mean response for each of the 32 vignettes, as well as the distribution of categorised responses (TKR, UKR or undecided). Notably, in 9% of vignettes (3 out of 32), surgeons did not reach a majority decision: no single option (TKR, UKR or undecided) was selected by >50% of participants. In nearly half of the vignettes (41%, 13 out of 32), the majority was slim (selected by 51%–55% of participants), demonstrating low consensus in TKR/UKR decision‐making.

**Table 5:** Responses per vignette.

Vignette	Response Mean	Responses categorised into ‘decisions’:		
		TKR	Undecided	UKR
		Count (%)	Count (%)	Count (%)
1	3.23	7 (8%)	5 (6%)	71 (86%)
2	3.23	9 (11%)	3 (4%)	71 (86%)
3	−0.08	38 (46%)	7 (8%)	38 (46%)
4	−0.10	44 (53%)	1 (1%)	38 (46%)
5	3.39	2 (2%)	9 (11%)	72 (87%)
6	3.20	8 (10%)	4 (5%)	71 (86%)
7	3.28	3 (4%)	7 (8%)	73 (88%)
8	−0.39	42 (51%)	2 (2%)	39 (47%)
9	0.39	37 (45%)	3 (4%)	43 (52%)
10	2.36	20 (24%)	2 (2%)	61 (73%)
11	2.28	13 (16%)	6 (7%)	64 (77%)
12	2.43	15 (18%)	2 (2%)	66 (80%)
13	2.80	11 (13%)	4 (5%)	68 (82%)
14	−1.08	51 (61%)	3 (4%)	29 (35%)
15	−0.75	42 (51%)	10 (12%)	31 (37%)
16	3.25	5 (6%)	7 (8%)	71 (86%)
17	0.27	31 (37%)	12 (14%)	40 (48%)
18	0.60	33 (40%)	3 (4%)	47 (57%)
19	1.30	21 (25%)	12 (14%)	50 (60%)
20	0.25	37 (45%)	1 (1%)	45 (54%)
21	−1.33	52 (63%)	5 (6%)	26 (31%)
22	−1.88	58 (70%)	3 (4%)	22 (27%)
23	0.39	36 (43%)	7 (8%)	40 (48%)
24	0.30	35 (42%)	2 (2%)	46 (55%)
25	0.77	24 (29%)	15 (18%)	44 (53%)
26	−2.72	66 (80%)	3 (4%)	14 (17%)
27	−1.25	51 (61%)	5 (6%)	27 (33%)
28	0.53	36 (43%)	2 (2%)	45 (54%)
29	1.54	19 (23%)	7 (8%)	57 (69%)
30	−0.23	42 (51%)	1 (1%)	40 (48%)
31	0.39	30 (36%)	10 (12%)	43 (52%)
32	−2.34	61 (73%)	6 (7%)	16 (19%)

*Note*: Blue shading indicates no consensus: surgeons did not reach a majority decision (TKR, UKR or undecided). Yellow shading indicates low consensus, with <55% of surgeons selecting the ‘majority’ decision.

Abbreviations: TKR, total knee replacement; UKR, unicompartmental knee replacement.

### Influence of clinical features on surgical judgements

When we regressed responses on the five manipulated clinical features, we found that preference for UKR was significantly lower for patients over 50 years (*b* = −0.57 [−0.82 to −0.33], *p* < 0.001), obese (BMI ≥ 30: *b* = −0.31 [−0.56 to −0.07], *p* = 0.012), with severe systemic disease (*b* = −0.46 [−0.70 to −0.21], *p* < 0.001) and/or abnormal ACL (*b* = −1.93 [−2.17 to −1.68], *p* < 0.001). Site of pain (generalised vs. medial) did not significantly influence TKR/UKR responses (*b* = −0.18 [−0.43 to 0.06], *p* = 0.144).

### Influence of surgeon factors on surgical judgements

The findings above remained robust when surgeon variables were added to the model (Table 6). Habitual practice (the tendency to perform UKR vs. TKR) was the only surgeon variable predictive of UKR preference (*b* = 1.26 [0.54–1.99], *p* = 0.001), indeed more predictive than most of the clinical factors investigated. Proportion of private work, AOT scores, fellowship training in UKR, and years of experience did not significantly impact TKR/UKR responses.

**Table 6:** Effect of clinical and surgeon factors on TKR/UKR responses (linear regression).

	*b* coefficient [95% CI], *p*
	A positive coefficient indicates that the factor increases preference for UKR A negative coefficient indicates that the factor decreases preference for UKR
**Clinical factors**	
Patient ACL integrity*	−1.93 [−2.17 to −1.68], *p* < 0.001
0 = normal	
1 = abnormal	
Patient age*	−0.57 [−0.82 to −0.33], *p* < 0.001
0 = below 50 years	
1 = equal to/above 50 years	
Patient ASA score*	−0.46 [−0.70 to −0.21], *p* < 0.001
0 = healthy/mild systemic disease	
1 = severe systemic disease	
Patient BMI*	−0.31 [−0.56 to −0.07], *p* = 0.012
0 = below 30	
1 = equal to/above 30	
Patient site of pain	−0.18 [−0.43 to 0.06], *p* = 0.144
0 = generalised	
1 = medial	
**Surgeon factors**	
Habitual practice*	1.26 [0.54–1.99], *p* = 0.001
0 = below median of 23% UKR	
1 = equal to/above median of 23% UKR	
% of work conducted privately	0.78 [−0.10 to 1.66], *p* = 0.082
0 = below median of 20%	
1 = equal to/above median of 20%	
AOT score	0.21 [−0.50 to 0.93], *p* = 0.559
0 = below median of 45	
1 = equal to/above median of 45	
Fellowship trained in UKR	−0.02 [−0.84 to 0.80], *p* = 0.967
0 = no	
1 = yes	
Years of experience	0.16 [−0.71 to 1.02], *p* = 0.725
0 = below median of 12 years	
1 = equal to/above median of 12 years	
Intercept	1.20 [0.18–2.23], *p* = 0.022

Abbreviations: ACL, anterior cruciate ligament; ASA, American Society of Anaesthesiologists; BMI, body mass index; CI, confidence interval; TKR, total knee replacement; UKR, unicompartmental knee replacement.

* Denotes predictors with statistical significance at *p* < 0.05.

### Influence of clinical features and surgeon factors on dichotomised responses (TKR vs. UKR)

The odds of choosing TKR over UKR increased by 37% on average when patients had severe systemic disease, 59% when they were aged over 50 years, and 258% when the ACL was abnormal (Table 7). BMI was not a significant predictor in this model, at *p* = 0.050. A greater tendency to perform UKR in practice reduced the odds of choosing TKR by 58% on average. No other variables significantly influenced dichotomised TKR/UKR responses.

**Table 7:** Effect of clinical and surgeon factors on dichotomised TKR/UKR responses (binary logistic regression).

	Odds ratio [95% CI], *p*
	An odds ratio >1 indicates that the factor increased the odds of choosing TKR An odds ratio <1 indicates that the factor decreased the odds of choosing TKR
**Clinical factors**	
Patient ACL integrity*	3.58 [2.96–4.34], *p* < 0.001
0 = normal	
1 = abnormal	
Patient age*	1.59 [1.32–1.92], *p* < 0.001
0 = below 50 years	
1 = equal to/above 50 years	
Patient ASA score*	1.37 [1.13–1.65], *p* = 0.001
0 = healthy/mild systemic disease	
1 = severe systemic disease	
Patient BMI	1.20 [1.00–1.45], *p* = 0.050
0 = below 30	
1 = equal to/above 30	
Patient site of pain	1.06 [0.88–1.28], *p* = 0.539
0 = generalised	
1 = medial	
**Surgeon factors**	
Tendency to perform UKR vs. TKR*	0.42 [0.25–0.72], *p* = 0.001
0 = below median of 23% UKR	
1 = equal to/above median of 23% UKR	
% of work conducted privately	0.54 [0.28–1.02], *p* = 0.056
0 = below median of 20%	
1 = equal to/above median of 20%	
AOT score	0.90 [0.54–1.52], *p* = 0.700
0 = below median of 45	
1 = equal to/above median of 45	
Fellowship trained in UKR	1.03 [0.57–1.87], *p* = 0.918
0 = no	
1 = yes	
Years of experience	0.90 [0.48–1.70], *p* = 0.755
0 = below median of 12 years	
1 = equal to/above median of 12 years	
Intercept	0.40 [0.19–0.85], *p* = 0.017

Abbreviations: ACL, anterior cruciate ligament; AOT, actively open‐minded thinking; ASA, American Society of Anaesthesiologists; BMI, body mass index; CI, confidence interval; TKR, total knee replacement; UKR, unicompartmental knee replacement.

* Denotes predictors with statistical significance at *p* < 0.05.

## DISCUSSION

Surgical decision‐making is a complex process influenced by personal experience, scientific evidence and guidelines [15]. A conscious, analytical, and deductive approach based predominantly on scientific evidence is complemented by an intuitive approach based on experience [8, 22]. Thus, understanding surgeons' reasoning in the context of clinical decision‐making is essential for both assessing competency in training and revalidation to ensure that surgeons are safe and continue to develop a reflective practice as new information or scientific evidence becomes available.

Previous studies have shown that experienced surgeons rely more on pattern recognition than effortful deliberation compared to novice surgeons in training [33]. Investigating the influence of both radiographic factors and patient demographics on how experienced arthroplasty surgeons decide whether to perform a UKR or TKR adds to the knowledge base of human decision‐making as well as potential future applications utilising artificial intelligence to augment human decision‐making [27].

This study used vignettes to elucidate the factors that UK surgeons attend to when deciding between a UKR or a TKR. We found that three clinical features mainly influenced TKR/UKR preference: ACL integrity was weighted heaviest, followed by age, and ASA score. BMI also seemed to exert some influence, but this was weak and unreliable in the data. We did not detect any influence of site of pain, which may indicate a recognition by surgeons that the location of knee pain can be diffuse and influenced by neuropathic symptoms [43].

The second strongest predictor of TKR or UKR preference, after ACL integrity, was nevertheless unrelated to patient features; it was the surgeons' habitual practice, that is, how much UKR versus TKR they tended to perform in real life. This indicates the profound effect of training, prior experience, and habit on clinical decision‐making. Future studies of implant registries may show a Kuhnian paradigm shift as older surgeons favouring the more traditional choice of TKR retire and younger surgeons trained in UKR influence implant choice in registries and studies [24].

The finding that surgeon‐related factors can surpass clinical characteristics in determining surgical choices offers valuable insight into the variability of surgical decision‐making, much of which may be driven by habit. Given that UKR has been reported to have a safer risk profile compared to TKR [7], understanding the determinants of implant choice is crucial, as they have significant implications for patient outcomes. Previous studies have primarily investigated patient factors and their impact on surgical decisions [3]. There remains a relative paucity of research on the role of surgeon‐specific factors and their influence on decision‐making.

The lack of a relationship between AOT scores and surgical decision‐making is also noteworthy. We hypothesised that higher AOT scores would correlate with a greater inclination toward UKR, assuming a tendency of open‐minded individuals to challenge the status quo. We found no evidence for this, though it is important to acknowledge that our study may not have been adequately powered to detect such subtle effects. Future research with larger, purposive samples could provide a deeper understanding of the cognitive styles influencing surgical choice.

We identified an inconsistency between the operation favoured in practice (TKR) and the operation favoured in our vignettes (UKR). This suggests that other factors (not captured in our vignettes) may come into consideration in real life. These could include organisational factors (e.g., incentives and constraints) and training. The discrepancy highlights the need to address the broader context in which surgical decisions are made. It suggests that clinical guidelines and decision‐support tools should consider not only patient characteristics but also the various external factors that can influence decision‐making in real‐life settings. More research is needed to identify and understand these contextual factors, which could provide valuable insights into how to better support surgeons in making informed, patient‐centred choices that align with both clinical evidence and practical realities.

ACL integrity was weighted heaviest in TKR/UKR decision‐making, far outweighing the influence of other clinical factors. Future work could explore the possible negative outcomes of prioritising ACL in this way. For example, are there any risks to weighting ACL integrity more heavily than age and ASA [5, 21, 30, 39]? Future work to determine the influence of a combination of factors such as implant design, age, obesity and ACL status could also consider surgeon choice and ability to perform a combined ACL reconstruction and UKR over a TKR within the complex surgical decision‐making process [47]. More recent studies have shown good results with combined ACL reconstruction and UKR, both with staged or simultaneous ACL reconstruction, with no difference in functional scores between those and patients with intact ACL [20].

Although the influence of BMI was weak in our data, it still merits discussion. Traditional exclusion criteria for UKR included obesity as a factor in favouring TKR, but a recent systematic review of 22 eligible studies showed that BMI > 30 is not a contra‐indication for UKR. Nevertheless, obese patients had a higher risk of aseptic failure and lower improvement in clinical scores compared to non‐obese patients, which may shift surgeon preference towards TKR [6]. Surgeon‐level data are available in the United Kingdom in the National Joint Registry; surgeons wishing to ensure their revision rates are low may therefore choose the ‘safer option’ in this patient group.

## LIMITATIONS

While clinical vignettes provide a high level of internal validity and experimental control, they do have the limitation of potentially lacking generalisability to real‐world settings. They simplify complex scenarios and may not fully encapsulate the multifaceted nature of clinical decision‐making. They measure how individuals tend to respond and cannot predict exactly how they would respond in the same situation, if it were encountered in real life. Nonetheless, studies have shown that responses to clinical vignettes align closely with those obtained under more realistic conditions [37, 38], suggesting that vignettes are a valid, reliable and cost‐effective proxy for real‐world clinical behaviour [12, 44]. They are also recognised as an effective measure for capturing variations in surgical judgement [18, 29].

We had to be selective in the factors that we chose to manipulate, as varying all relevant factors would have resulted in a prohibitively large set of vignettes. Notably, patient preference was kept constant (the patient has no preference), as the study was intended to focus on surgeons' rather than patients' preferences. We acknowledge that, in reality, factors such as return to work, speed of recovery, revision, complications and functional outcomes are key considerations for patients [48]. Failure to include these factors therefore limits the generalisability of our findings to real‐life settings, but also presents an important avenue for future research.

We did not explore factors such as patient gender or ethnicity, which could potentially influence decision‐making. Conducted solely in the United Kingdom, the study's applicability to other healthcare systems with differing surgical guidelines, practices, and cultures may be limited.

The majority of arthroplasty surgeons in the United Kingdom are male; yet studies have shown a difference in how male and female surgeons process information. A study found male physicians to make quicker, more intuitive decisions, whereas female physicians were more comprehensive and took longer to evaluate information [14]. Patient‐surgeon sex discordance may also affect decision‐making with preliminary studies in hip surgery showing potential impacts on patient safety [13]. Further research is needed to determine the impact of patient–surgeon sex discordance and decision‐making.

A larger multicentre study exploring further factors in surgeon decision‐making, such as surgeon–patient sex discordance, ethnicity, socioeconomic group, obesity, age and ACL integrity together with implant choice, would provide the answer to some of these questions.

## CONCLUSION

Surgeons relied mainly on three clinical factors to determine their preference between TKR and UKR: ACL integrity (weighted heaviest), age and ASA score. The strongest surgeon determinant of UKR/TKR preference was habitual practice. Overall, surgeons performed more TKRs in practice but showed a somewhat greater preference for UKR in our study, suggesting that other factors (not studied here) might influence real‐world decision‐making. Replicating the study with a different surgeon cohort could enhance the robustness of our findings.

## AUTHOR CONTRIBUTIONS

Martine Nurek, Caroline B. Hing, Justin Cobb and Olga Kostopoulou conceived the idea and designed the study. Martine Nurek, Caroline B. Hing, Justin Cobb and Olga Kostopoulou designed the preliminary survey. Martine Nurek, Omar Musbahi, Martinique Vella Baldacchino, Caroline B. Hing, Justin Cobb and Olga Kostopoulou constructed all study materials. Martine Nurek put the study materials on the Qualtrics platform, collected and analysed the data, in discussions with Olga Kostopoulou. Robert Hamm contributed to data analysis. Martine Nurek and Omar Musbahi wrote the first draft of the manuscript, with critical input from Caroline B. Hing, Olga Kostopoulou and UNITES members. All authors approved the submitted manuscript.

## UNITES CONSORTIUM

All members of the UNITES Consortium are also acknowledged for supporting this research: Barry Andrews Mb ChB BMed Sci FRCSEd; Tony Smith FRCS (Orth); P. J. Walmsley MD (Res) FRCS (Orth); David W. Elson MBChB, MRCS, FRCS; Alex Anderson MBBS MRCS (Ed) FRCS (Orth); Chloe E. H. Scott FRCSEd (Tr&Orth) MSc MD; Alasdair J. A. Santini FRCS (Orth); Benedict J. A. Lankester BM BCh MRCS FRCS (Tr&Orth); J. William Tice MA FRCS; Reza Mansouri MD (Hons) FRCS (Tr&Orth) MFSEM (UK); Arjuna Imbuldeniya B.Med.Sci, MSc,DIC,FRCS (Tr&Orth); Tarek Boutefnouchet MBChB, PGCert Med Ed, MSc (Tr&Orth), FRCS (Tr&Orth), Dipl. Football Med.; Nathanael Ahearn MSc, FRCS (Tr&Orth); Stefan Bajada MD FRCS (Tr&Orth) PhD; James R. A. Smith MBBS BSc FRCS (Tr&Orth); Pregash Ellapparadja FRCS (T&O); Alan R. Norrish MBA, LLM, PhD, FRCS (Orth); Sean O'Leary FRCS (Tr&Orth); Jon Campion FRCS (London); Benjamin V. Bloch BSc MBBS FRCS (Tr&Orth); Ricardo J. Pacheco FRCS (Tr&Orth); Simon B. Barton MBChB, FRCS, MSC SEM; Adrian Cassar‐Gheiti, Consultant Orthopaedic; David Selvan MBChB (Hon), FRCS (Tr&Orth), MFSTEd; Jonathan Phillips FRCS (Tr&Orth); Sushrut Kulkarni FRCS (Tr&Orth); Randeep S. Aujla MBChB ChM FRCS MFSEM; Philip G. Turner MB ChB, FRCSEd, FRCSEng, FFSEM; Amit Patel FRCS (Orth); Rahul S. Kotwal MS, FRCS (Tr&Orth); Ashim Mannan MBBS, BsC, FRCS (Tr&Orth); Rahul Bhattacharyya MBCHB (Hons), MD (Res), FRCS (Orth), MSc, MRCS; Ahmed Mabrouk MBBCH (Hons), MRCS, FRCS (Tr&Orth); Aaron Biing Yann Ng FRCS (Tr&Orth); Keshav Mathur Ms (Orth), FRCS (Orth); Muhammad Adeel Akhtar BSc, MBBS, MRCSEd, Dip SEM (UK), PG Dip CAOS, MD (Res), MFSEM, MFSTEd, MFCI, FEBOT, FFSTEd, FRCSEd (Tr&Orth); Robert William Walker FRCS (Tr&Orth); Rakesh Kucheria MBBS, FRCS (Eng), FRCS (Tr&Orth); A. D. Liddle DPhil FRCS (Orth); Lebur Rohman FRCSed (Tr&Orth); Ravikumar Pydisetty MBBS, MS, DNB, FRCS, FRCS (Orth), MCh (liv); Manish Divekar MS Ortho, MCh Ortho, FRCS (Tr&Orth), PGCE; Manish Kiran MS (Orth), DNB, MCh (Orth), FRCS Ed (Tr&Orth); David Houlihan‐Burne MB BS (Hons), BSC (Hons), MRCS, FRCS (Eng); N. D. Rossiter FRCSEd (Tr&Orth), FFSTEd; Sanjeev Agarwal FRCS (Orth); N. J. London MA MD (Cantab.) FRCS (Tr&Orth); AD Toms MB.ChB. FRCS (Orth) MSc; Zuhair Nawaz FRCS (Tr&Orth); Phil Hopgood FRCS; Ghias Bhattee MBBS, MRCS, FRCS (Tr&Orth); Moez Zeiton MBChB MSc MRCS FRCS (Tr&Orth); Khalid 2Al‐Dadah FRCS (Tr&Orth); Hywel Davies MBBS MSc FRCS (Tr&Orth); Oliver S Schindler BSc (Hons) PhD FMH MFSEM FRCSEng FRCS(Orth); H. B. Waterson FRCS MD; SP White FRCS (Tr&Orth); A. J. Kelly MA FRCS (Orth); Yuvraj Agrawal MB BS, FRCS (Tr&Orth); Christopher Wilson FRCS (Orth); Nicholas E. Ohly MSc, FRCSEd (Orth); Andrew Lavender FRCS (Tr&Orth); Morgan Bayley MB BCh, FRCS; Fazal Ali FRCS (Tr&Orth); Nivraj Singh Bhamber MBBS, BSc (Hons), FRCS (Tr&Orth); Tarique Parwez FRCS (Tr&Orth), FRCS, Diploma Orth Engineering; Christopher Buckle FRCS MSc (Oxon) BSc; Zameer Shah FRCS MBA; Jeremy Rushbrook MB ChB (Hons), MSc (Eng), FRCS (Tr&Orth); Damon Simmons FRCS FRCS (Orth); Amit Bishnoi FRCS (Tr&Orth); Reshid Berber MBBS, FRCS (Orth), PhD; Richard Parkinson FRCS (Orth); D. Prakash MBBS, MCh, FRCS (Ed), FRCS (Glas), FRCS (Tr&Orth); James R. D. Murray MA FRCS (Tr&Orth); Sujit Agarwal FRCS (Tr&Orth).

## CONFLICT OF INTEREST STATEMENT

The authors declare no conflicts of interest. However, some study participants and some of those in the collaborative UNITES consortium have royalties and/or are design surgeons for STRYKER, Zimmer Biomet and Arthrex.

## ETHICS STATEMENT

The preliminary survey and the main study were approved by the Imperial College Research Ethics Committee (ICREC), reference numbers 20IC6288 and 21IC7201, respectively.

## Supporting information

Supporting information.

## Data Availability

The dataset supporting the conclusions of this article is publicly available in the Open Science Framework: https://osf.io/wz3xf/.
