# Supplementary material for: Factors influencing UK arthroplasty surgeons' decision‐making between total and medial unicompartmental knee surgery: A vignette‐based behavioural experiment
Source: J Exp Orthop. 2025 Feb 28;12(1):e70178. doi: 10.1002/jeo2.70178 (PMC11869565; doi:10.1002/jeo2.70178)
Supplement: Supplementary file 1 — Supporting information. [file JEO2-12-e70178-s001.docx]

**Factors influencing UK arthroplasty surgeons’ decision making between total and medial unicompartmental knee surgery: A vignette-based behavioural experiment**

**SUPPLEMENTARY MATERIALS (SM)**

**SM1: The 32 clinical vignettes**

**Vignette 1:** Patient A (aged 47, BMI 26) presents with generalised knee pain. Their ASA Physical Classification Score is 1 (healthy). On examination, ACL is normal.

**
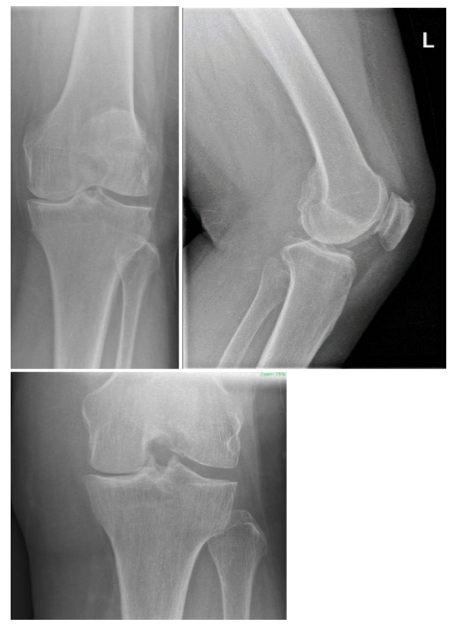
**

Vignette 2: Patient B (aged 60, BMI 29) presents with generalised knee pain. Their ASA Physical Classification Score is 2 (mild systemic disease). On examination, ACL is normal.


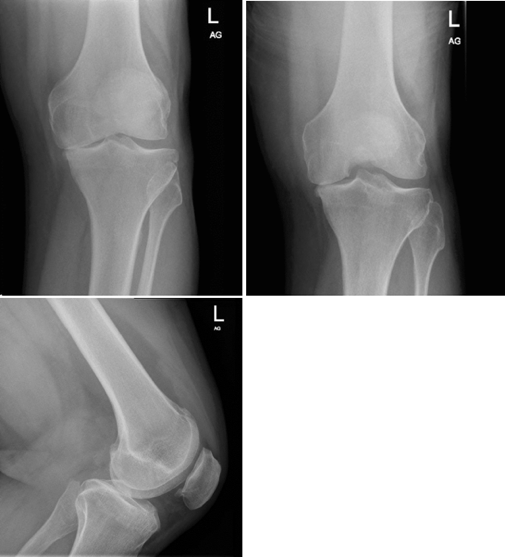


Vignette 3: Patient C (aged 44, BMI 30) presents with generalised knee pain. Their ASA Physical Classification Score is 1 (healthy). On examination, ACL is normal.


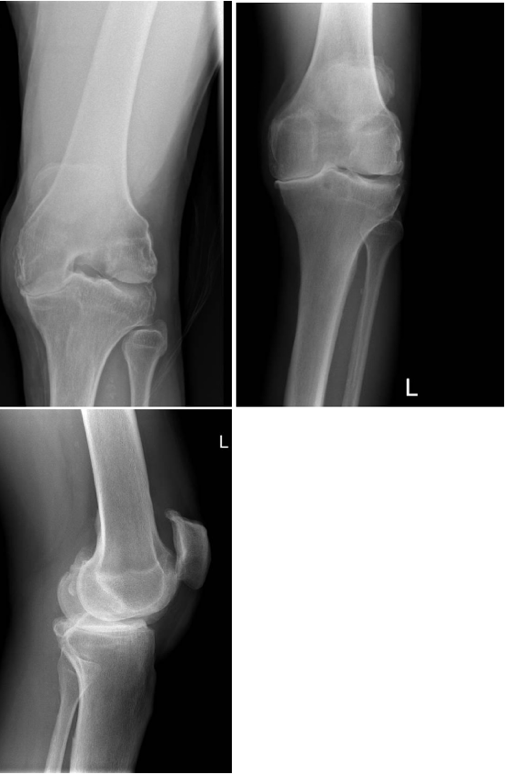


**Vignette 4:** Patient D (aged 56, BMI 31) presents with generalised knee pain. Their ASA Physical Classification Score is 2 (mild systemic disease). On examination, ACL is normal.


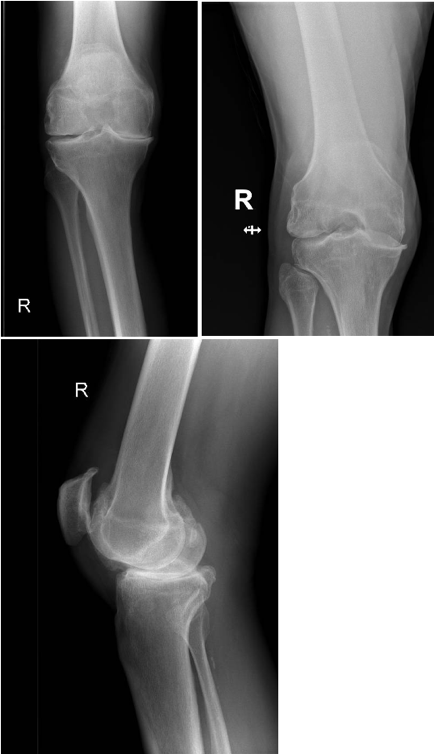


**Vignette 5:** Patient E (aged 40, BMI 21) presents with medial knee pain. Their ASA Physical Classification Score is 1 (healthy). On examination, ACL is normal.

**
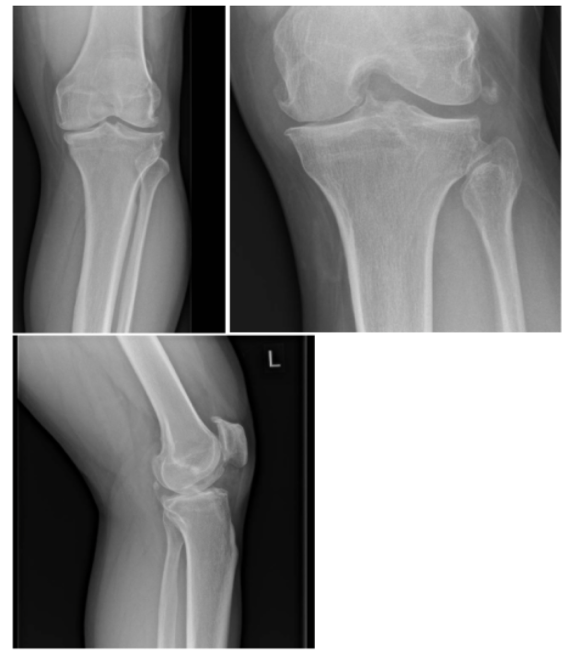
**

Vignette 6: Patient F (aged 63, BMI 24) presents with medial knee pain. Their ASA Physical Classification Score is 2 (mild systemic disease). On examination, ACL is normal.


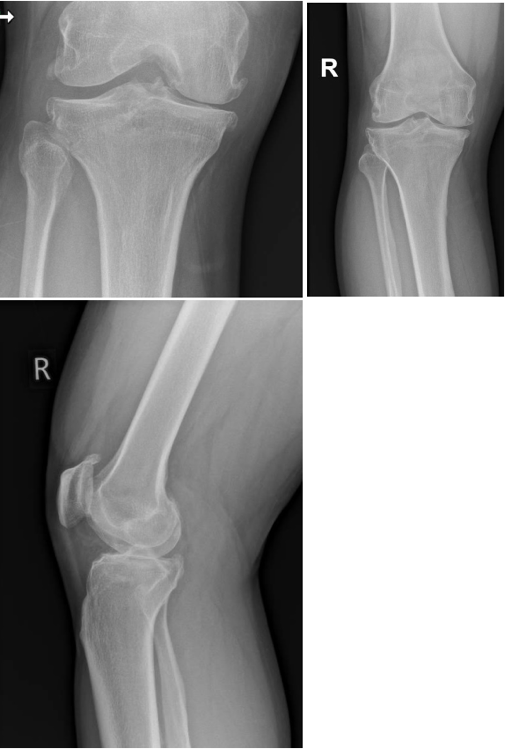


Vignette 7: Patient G (aged 48, BMI 33) presents with medial knee pain. Their ASA Physical Classification Score is 2 (mild systemic disease). On examination, ACL is normal.


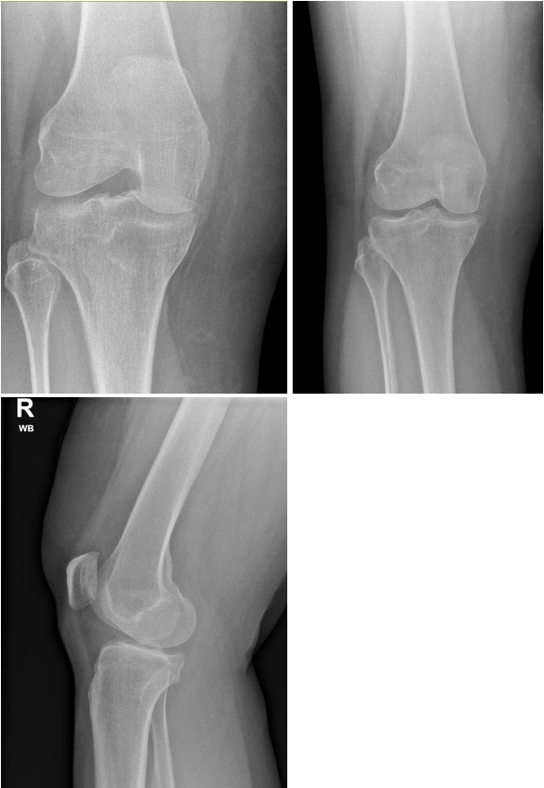


**Vignette 8:** Patient H (aged 80, BMI 31) presents with medial knee pain. Their ASA Physical Classification Score is 2 (mild systemic disease). On examination, ACL is normal.


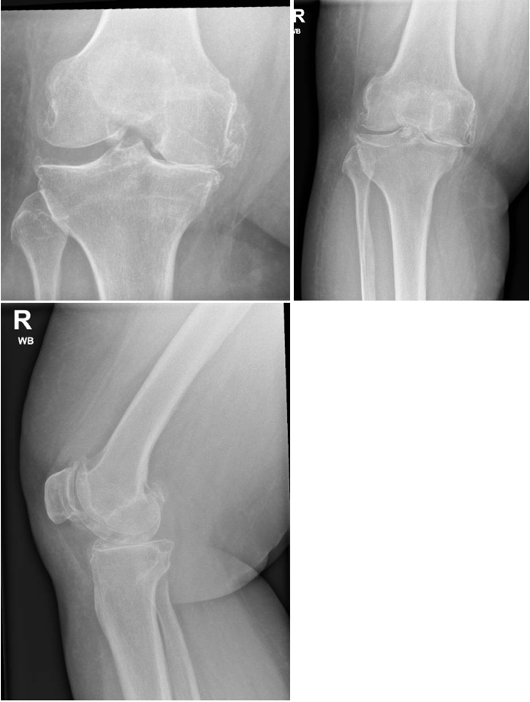


**Vignette 9:** Patient I (aged 49, BMI 22) presents with generalised knee pain. Their ASA Physical Classification Score is 3 (severe systemic disease). On examination, ACL is normal.
 

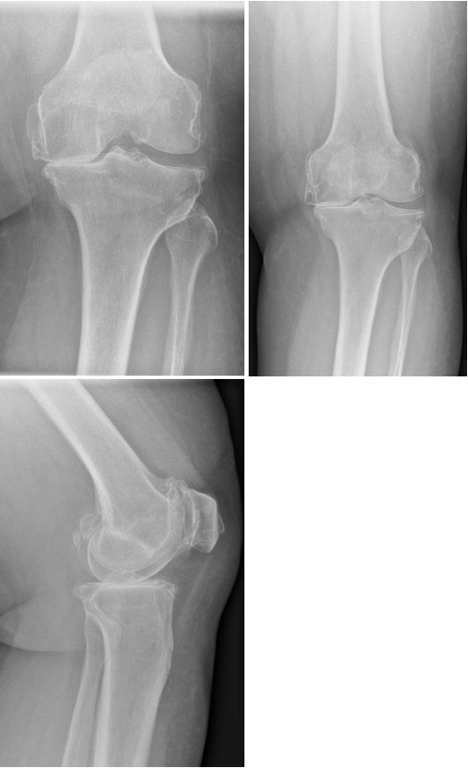


Vignette 10: Patient J (aged 77, BMI 27) presents with generalised knee pain. Their ASA Physical Classification Score is 3 (severe systemic disease). On examination, ACL is normal.


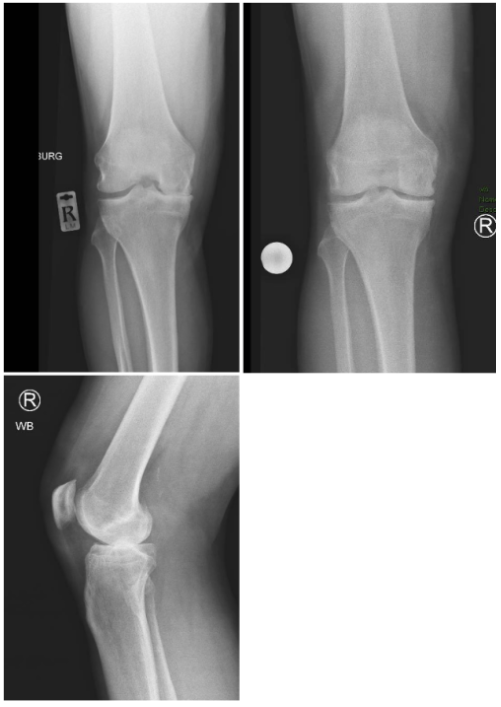


Vignette 11: Patient K (aged 45, BMI 32) presents with generalised knee pain. Their ASA Physical Classification Score is 3 (severe systemic disease). On examination, ACL is normal.


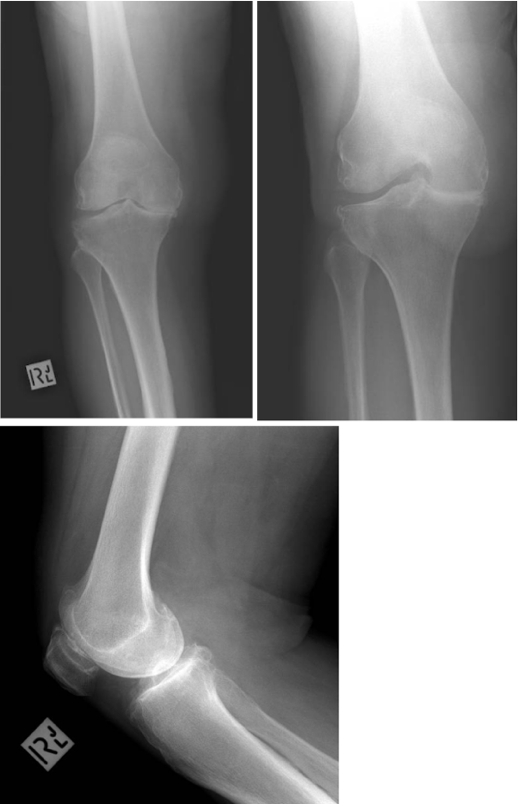


**Vignette 12:** Patient L (aged 54, BMI 33) presents with generalised knee pain. Their ASA Physical Classification Score is 3 (severe systemic disease). On examination, ACL is normal.

**
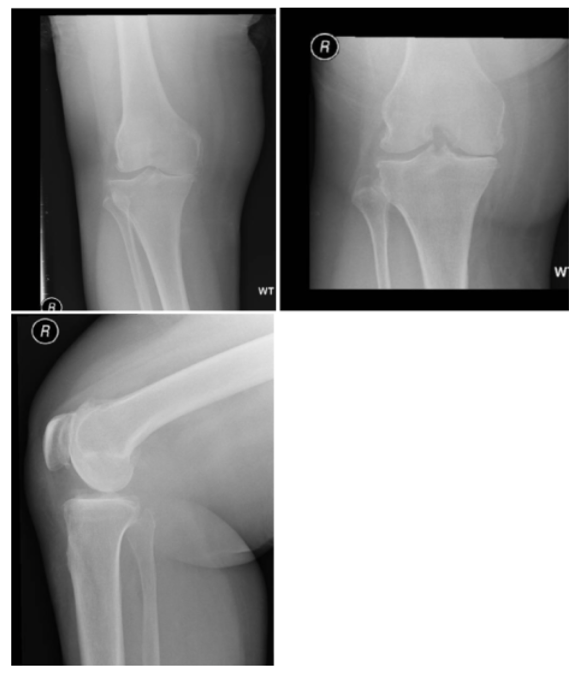
**

**Vignette 13:** Patient M (aged 46, BMI 23) presents with medial knee pain. Their ASA Physical Classification Score is 3 (severe systemic disease). On examination, ACL is normal.

**
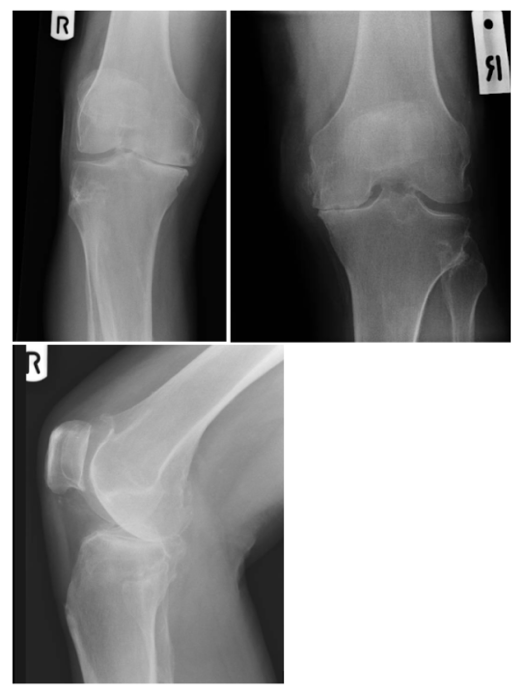
**

**Vignette 14:** Patient N (aged 71, BMI 26) presents with medial knee pain. Their ASA Physical Classification Score is 3 (severe systemic disease). On examination, ACL is normal.


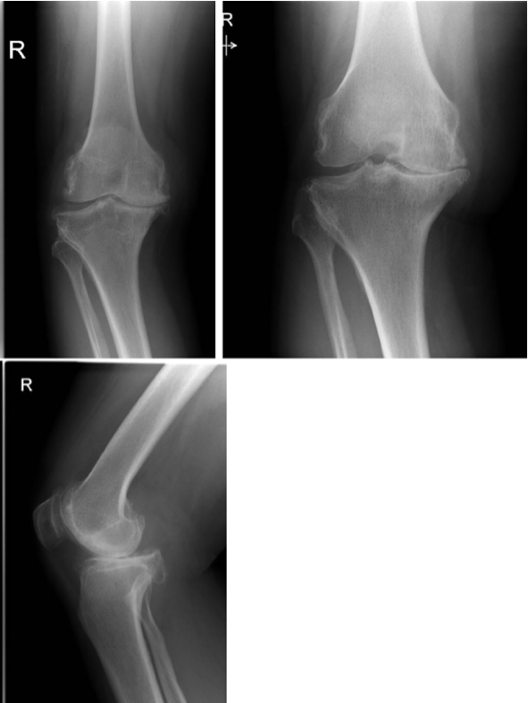


**Vignette 15:** Patient O (aged 40, BMI 35) presents with medial knee pain. Their ASA Physical Classification Score is 3 (severe systemic disease). On examination, ACL is normal.
 

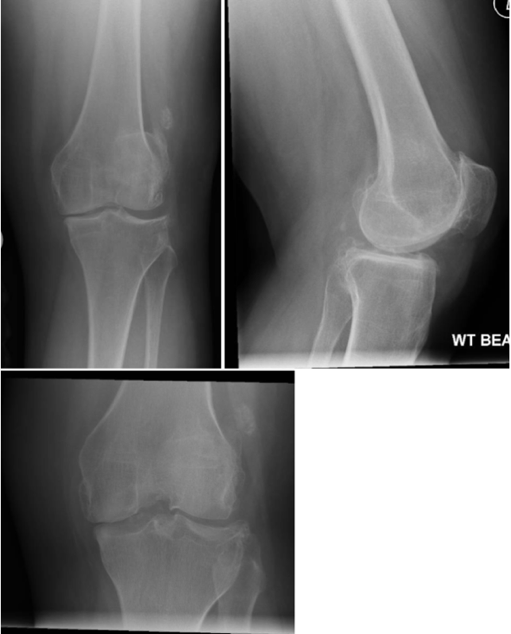


**Vignette 16:** Patient P (aged 65, BMI 34) presents with medial knee pain. Their ASA Physical Classification Score is 3 (severe systemic disease). On examination, ACL is normal.


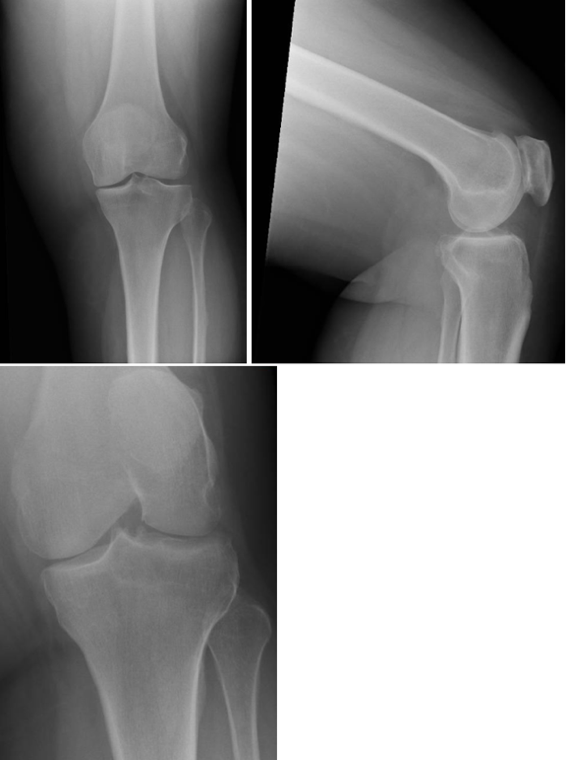


**Vignette 17:** Patient Q (aged 45, BMI 19) presents with generalised knee pain. Their ASA Physical Classification Score is 1 (healthy). On examination, ACL is abnormal.


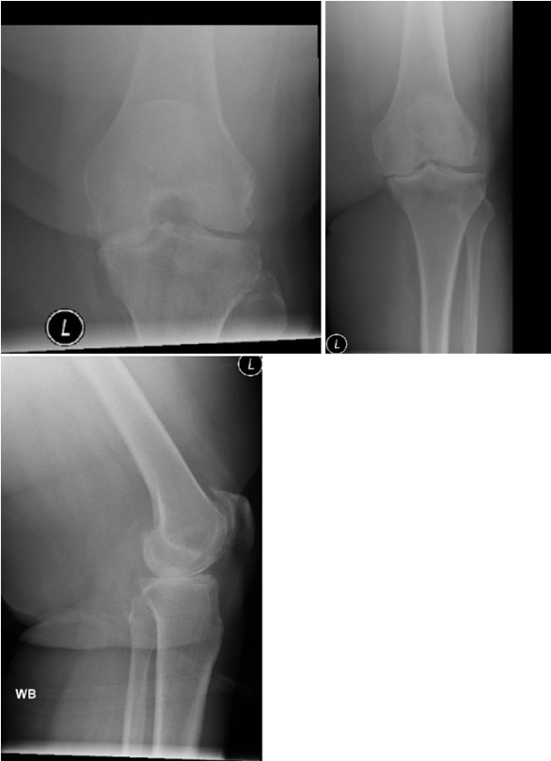


**Vignette 18:** Patient R (aged 50, BMI 20) presents with generalised knee pain. Their ASA Physical Classification Score is 1 (healthy). On examination, ACL is abnormal.

**
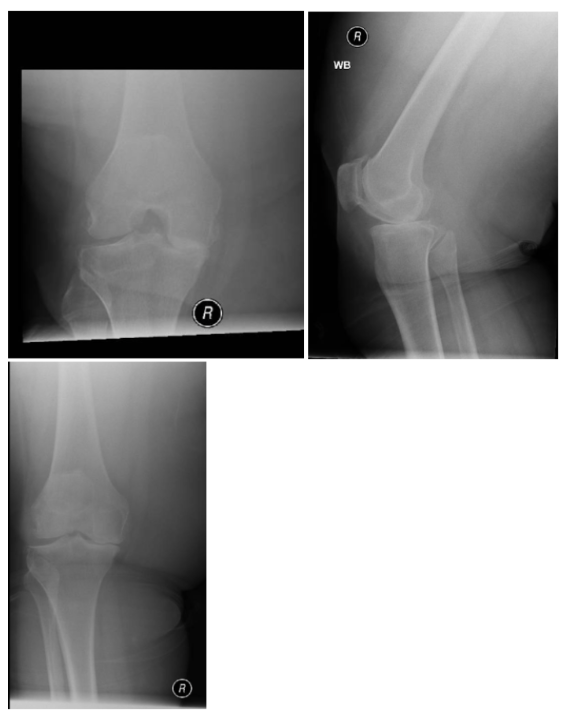
**

**Vignette 19:** Patient S (aged 45, BMI 32) presents with generalised knee pain. Their ASA Physical Classification Score is 1 (healthy). On examination, ACL is abnormal.


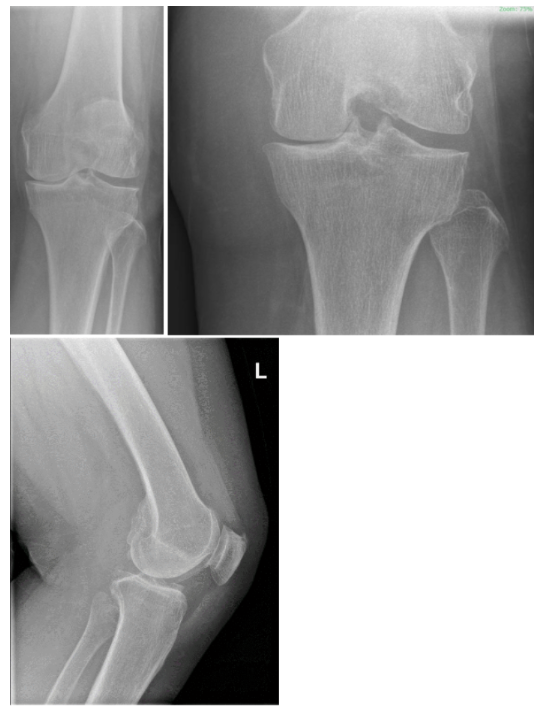


**Vignette 20:** Patient T (aged 75, BMI 33) presents with generalised knee pain. Their ASA Physical Classification Score is 2 (mild systemic disease). On examination, ACL is abnormal.


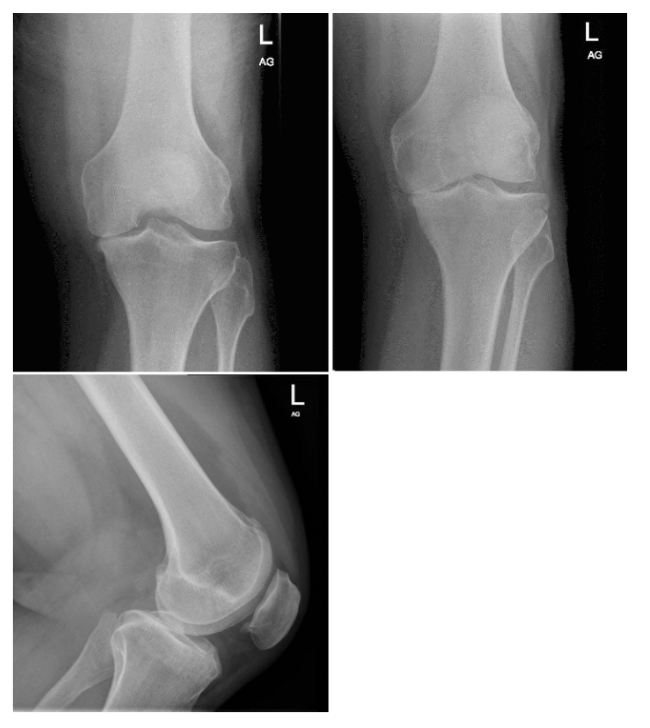


**Vignette 21:** Patient U (aged 49, BMI 23) presents with medial knee pain. Their ASA Physical Classification Score is 2 (mild systemic disease). On examination, ACL is abnormal.


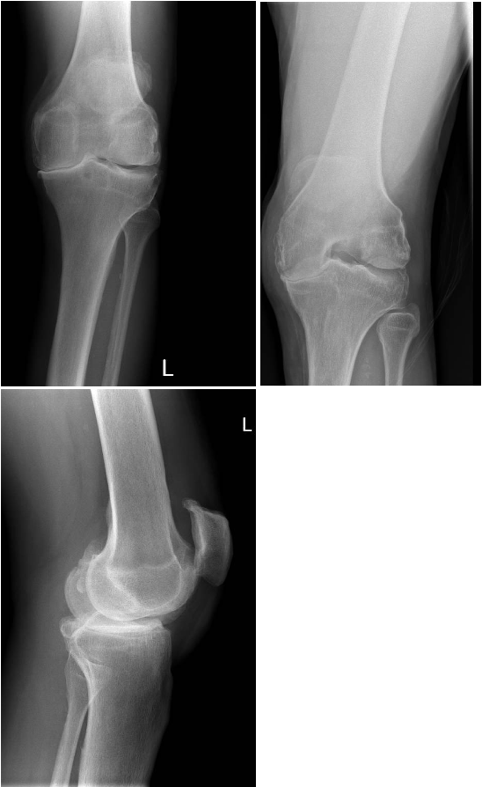


**Vignette 22:** Patient V (aged 68, BMI 29) presents with medial knee pain. Their ASA Physical Classification Score is 1 (healthy). On examination, ACL is abnormal.


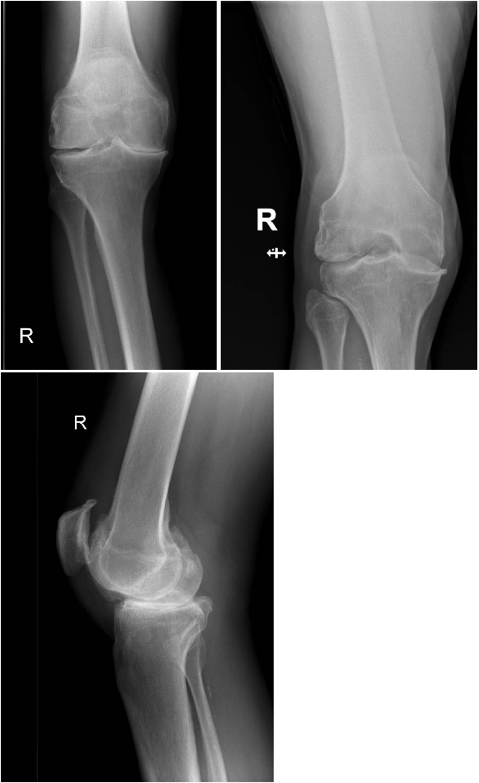


**Vignette 23:** Patient W (aged 47, BMI 35) presents with medial knee pain. Their ASA Physical Classification Score is 2 (mild systemic disease). On examination, ACL is abnormal.


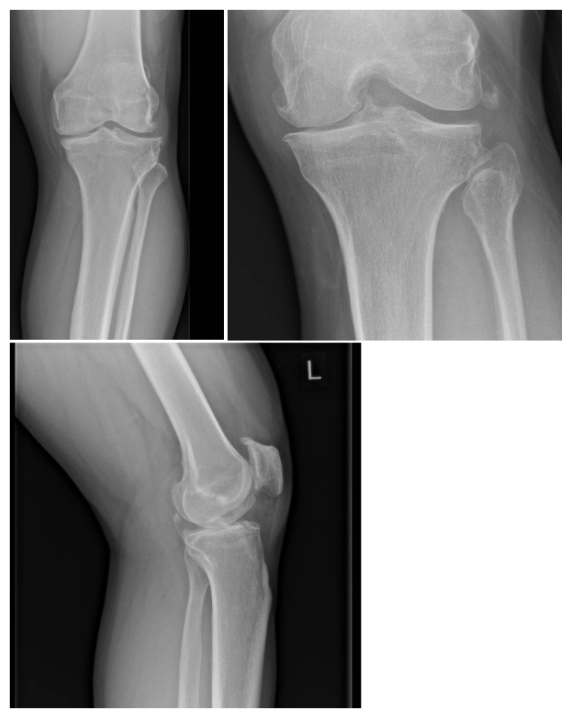


**Vignette 24:** Patient X (aged 66, BMI 30) presents with medial knee pain. Their ASA Physical Classification Score is 1 (healthy). On examination, ACL is abnormal.


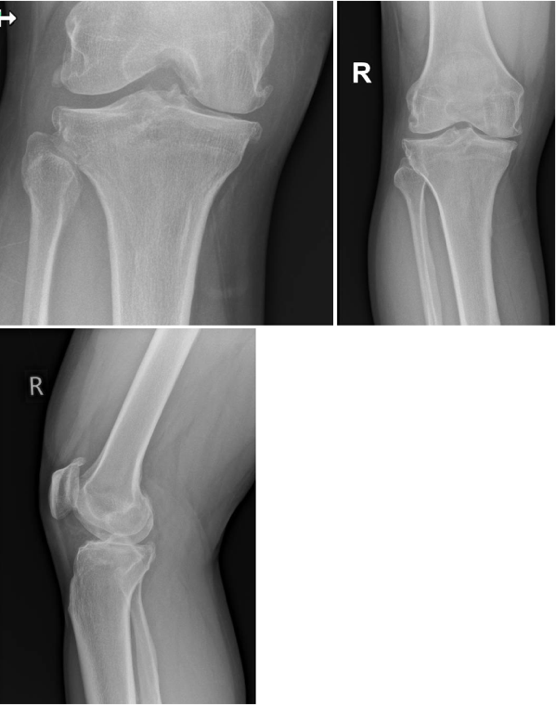


**Vignette 25:** Patient Y (aged 48, BMI 19) presents with generalised knee pain. Their ASA Physical Classification Score is 3 (severe systemic disease). On examination, ACL is abnormal.


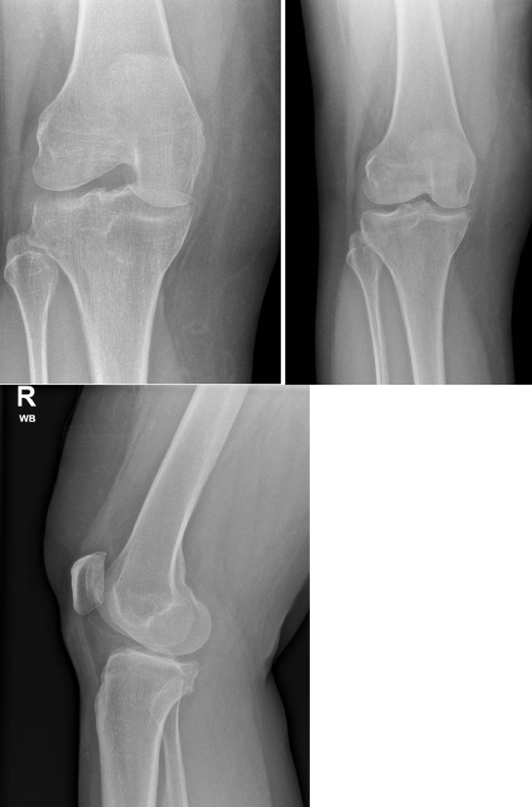


Vignette 26: Patient Z (aged 59, BMI 26) presents with generalised knee pain. Their ASA Physical Classification Score is 3 (severe systemic disease). On examination, ACL is abnormal.


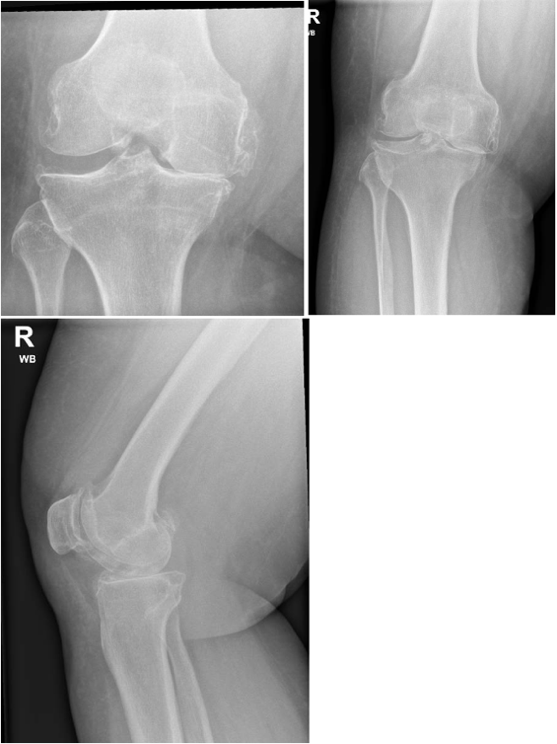


**Vignette 27:** Patient DW (aged 46, BMI 36) presents with generalised knee pain. Their ASA Physical Classification Score is 3 (severe systemic disease). On examination, ACL is abnormal.


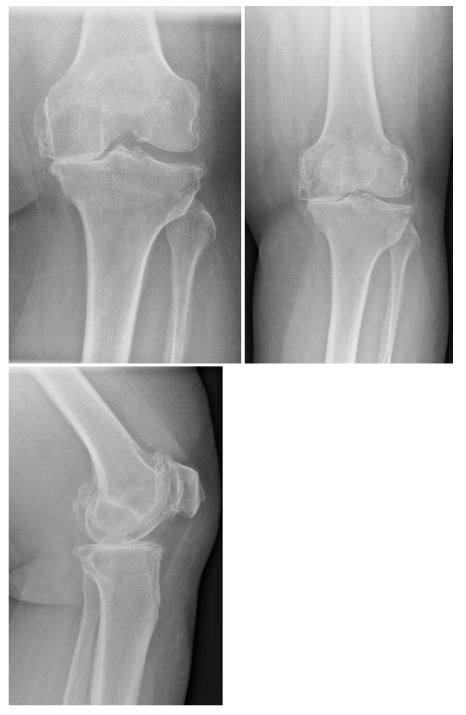


Vignette 28: Patient PL (aged 74, BMI 32) presents with generalised knee pain. Their ASA Physical Classification Score is 3 (severe systemic disease). On examination, ACL is abnormal.


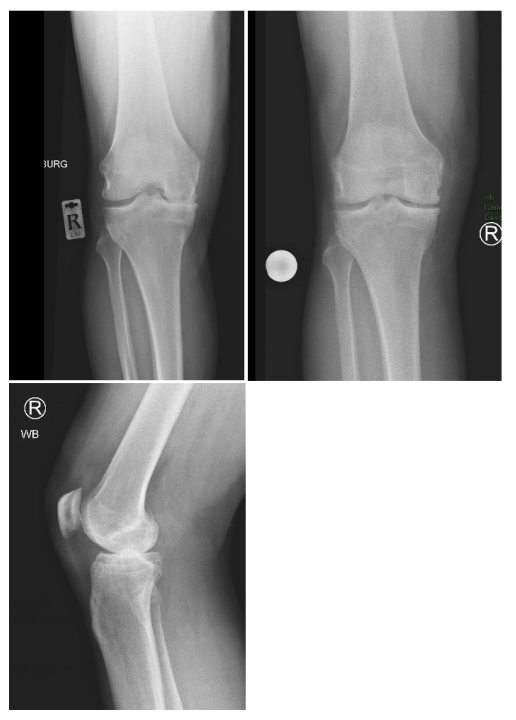


Vignette 29: Patient JS (aged 40, BMI 27) presents with medial knee pain. Their ASA Physical Classification Score is 3 (severe systemic disease). On examination, ACL is abnormal.


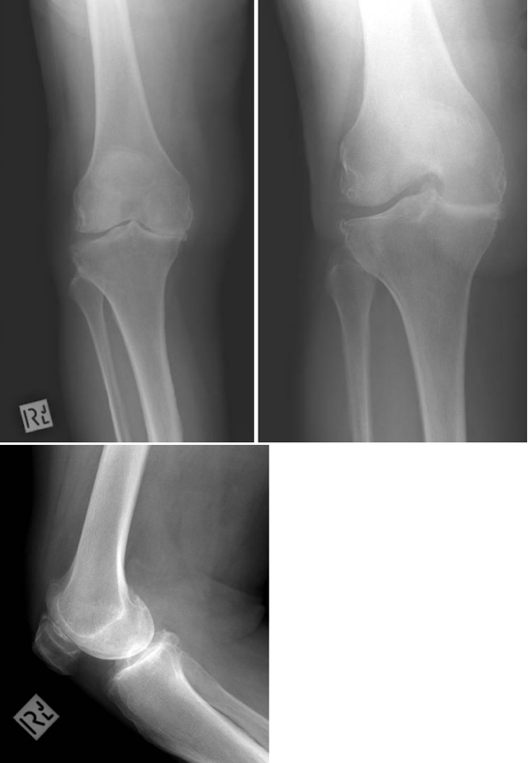


**Vignette 30:** Patient KS (aged 79, BMI 21) presents with medial knee pain. Their ASA Physical Classification Score is 3 (severe systemic disease). On examination, ACL is abnormal.


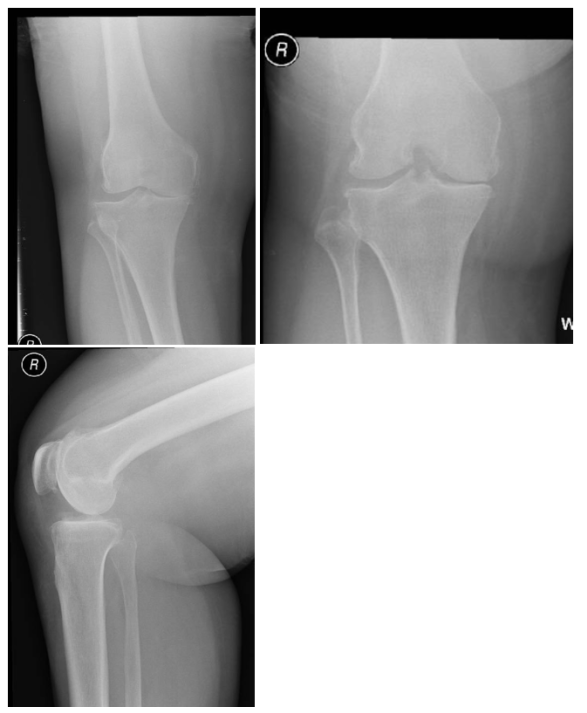


**Vignette 31:** Patient HW (aged 43, BMI 31) presents with medial knee pain. Their ASA Physical Classification Score is 3 (severe systemic disease). On examination, ACL is abnormal.


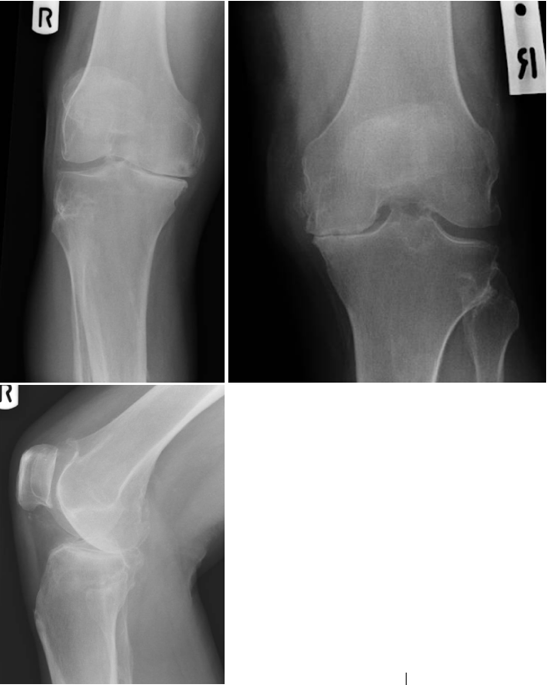


**Vignette 32:** Patient SM (aged 53, BMI 34) presents with medial knee pain. Their ASA Physical Classification Score is 3 (severe systemic disease). On examination, ACL is abnormal.


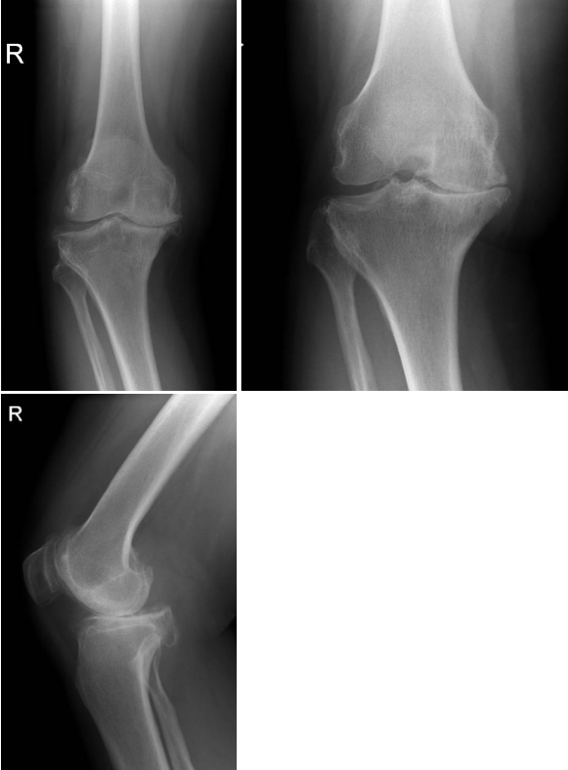


**SM2: Actively Open-minded Thinking (AOT) scale (Baron, 2019)**

1. Allowing oneself to be convinced by a solid opposing argument is a sign of good character.
2. People should take into consideration evidence that goes against conclusions they favour.
3. Being undecided or unsure is the result of muddled thinking (R).
4. People should revise their conclusions in response to relevant new information.
5. Changing your mind is a sign of weakness (R).
6. People should search actively for reasons why they might be wrong.
7. It is OK to ignore evidence against your established beliefs (R).
8. It is important to be loyal to your beliefs even when evidence is brought to bear against them (R).
9. When we are faced with a new question, the first answer that occurs to us is usually best (R).
10. Good thinking leads to uncertainty when there are good arguments on both sides.
11. When faced with a new question, we should consider more than one possible answer before reaching a conclusion.

For each statement, surgeons indicated their agreement on a 5-point scale, ranging from “*Completely disagree*” to “*Completely agree*” with midpoint “*Neutral*”. (R) = reverse score.
